# Ultrasound-guided transversus abdominis plane block as an adjunctive anesthesia technique in elderly patients with combined massive ascites: a case report and literature review

**DOI:** 10.3389/fmed.2025.1541462

**Published:** 2025-03-05

**Authors:** Liwen Zhang, Aihong Liu, Lei Wang, Yanping Zhang, Zhaolan Hu

**Affiliations:** ^1^Discipline of Anesthesiology, Qingdao Medical College, Qingdao University, Qingdao, China; ^2^Department of Gynecology, Qingdao Hiser Hospital Affiliated of Qingdao University (Qingdao Traditional Chinese Medicine Hospital), Qingdao, China; ^3^Department of Urology Surgery, Qingdao Hiser Hospital Affiliated of Qingdao University (Qingdao Traditional Chinese Medicine Hospital), Qingdao, China; ^4^Department of Anesthesiology, Qingdao Hiser Hospital Affiliated of Qingdao University (Qingdao Traditional Chinese Medicine Hospital), Qingdao, China

**Keywords:** ovarian tumor excision, transversus abdominal plane block, elderly, ascites, ultrasound, case report

## Abstract

The ultrasound-guided transversus abdominis plane (TAP) block has emerged as an effective adjunctive analgesic technique for abdominal surgery. However, its use in older patients with significant ascites has been rarely documented. This report presents the anesthetic management of an older patient with massive ascites undergoing open laparotomy for an ovarian tumor. Preoperatively, 30 mL of 0.2% levobupivacaine was injected into the TAP under ultrasound guidance. The procedure was uneventful, with approximately 9,000 mL of ascitic fluid drained, along with the removal of a 13 × 13 × 7-cm left ovarian mass, an 8 × 5.5 × 4-cm uterus, and a 3.5 × 1 × 0.5-cm right ovary. Throughout the surgery, the patient maintained hemodynamic stability, with no significant fluctuations in blood pressure or heart rate. Postoperatively, the patient reported minimal pain and experienced no adverse effects. These findings highlight the effectiveness of ultrasound-guided TAP block as an auxiliary anesthesia technique, providing enhanced analgesia, promoting hemodynamic stability, and improving overall anesthetic outcomes in older patients with substantial ascites.

## Introduction

The transversus abdominis plane (TAP) block, introduced by Rafi (1), is a regional anesthesia technique that targets the thoracolumbar nerves originating from the T6–L1 spinal roots, which traverse the plane between the internal oblique and transversus abdominis muscles (2). Injecting a local anesthetic into this plane allows for the blockade of nerve afferents, providing effective analgesia to the anterolateral abdominal wall. Advances in ultrasound technology have improved the precision and safety of TAP blocks, leading to their increasing adoption as an adjunctive analgesic technique for abdominal surgeries. Over the past decade, growing evidence has demonstrated the efficacy of TAP blocks in various abdominal procedures, underscoring their integral role in multimodal analgesia (3, 4).

Patients with ovarian tumors and associated ascites often present unique anesthetic challenges due to coexisting comorbidities such as anemia and hypoproteinemia. Massive ascites can exacerbate these conditions by elevating the diaphragm, reducing thoracic cavity volume, and impairing ventilation, which may lead to hypoxia and hypercapnia. In addition, the prolonged compression of the abdominal aorta and inferior vena cava by the tumor and ascites can reduce venous return, resulting in hypotension and tachycardia. These hemodynamic changes are further complicated by the opening of lower limb vascular beds and the activation of collateral circulatory pathways in abdominal organs. Therefore, anesthetic management in these patients must prioritize maintaining hemodynamic stability and fluid balance to mitigate the risk of complications. Insufficient fluid resuscitation can lead to significant hypotension and impaired organ perfusion, while excessive fluid administration or frequent use of vasoactive drugs may precipitate acute pulmonary edema by increasing preload. Therefore, selecting an appropriate anesthetic technique is critical for optimizing perioperative outcomes in this patient population.

**Figure 1:**
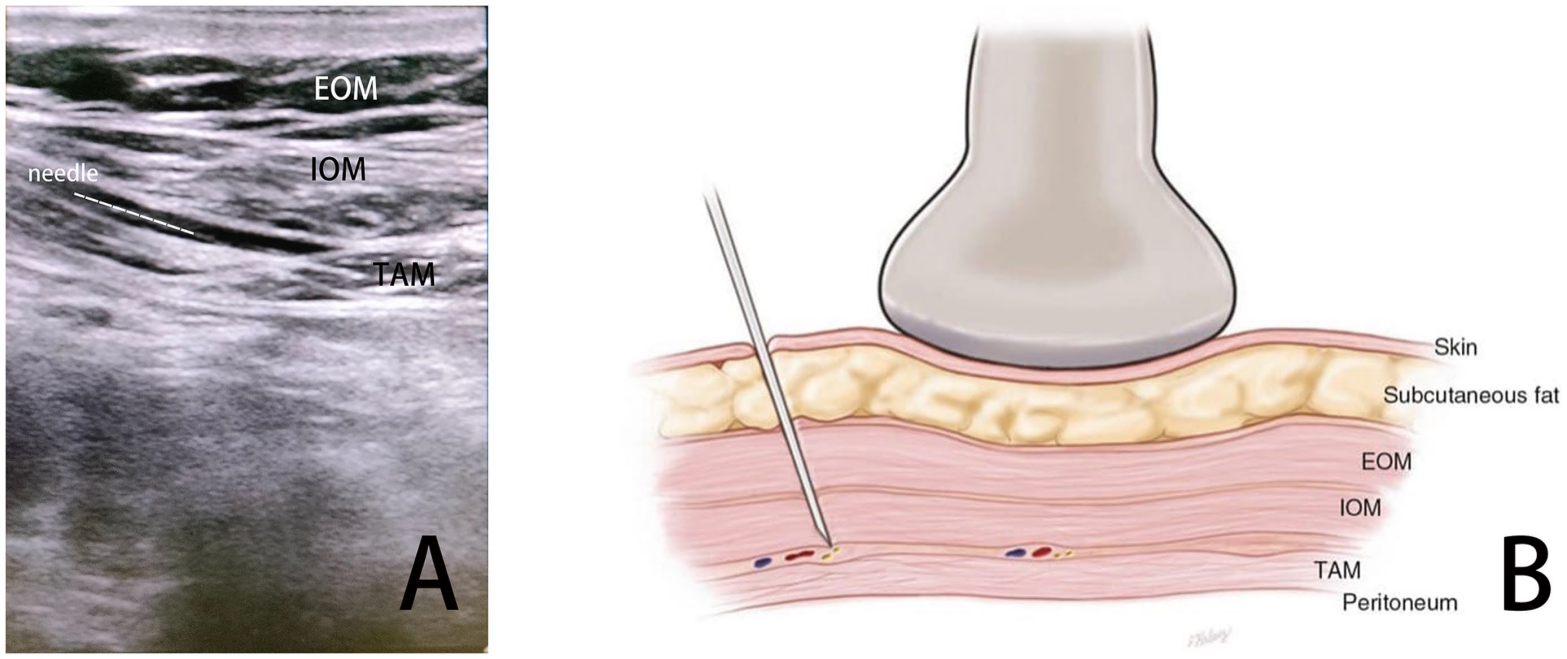
TAP block diagram. **(A)** Ultrasound imaging for TAP block. **(B)** Schematic representation of the TAP block.

**Figure 2:**
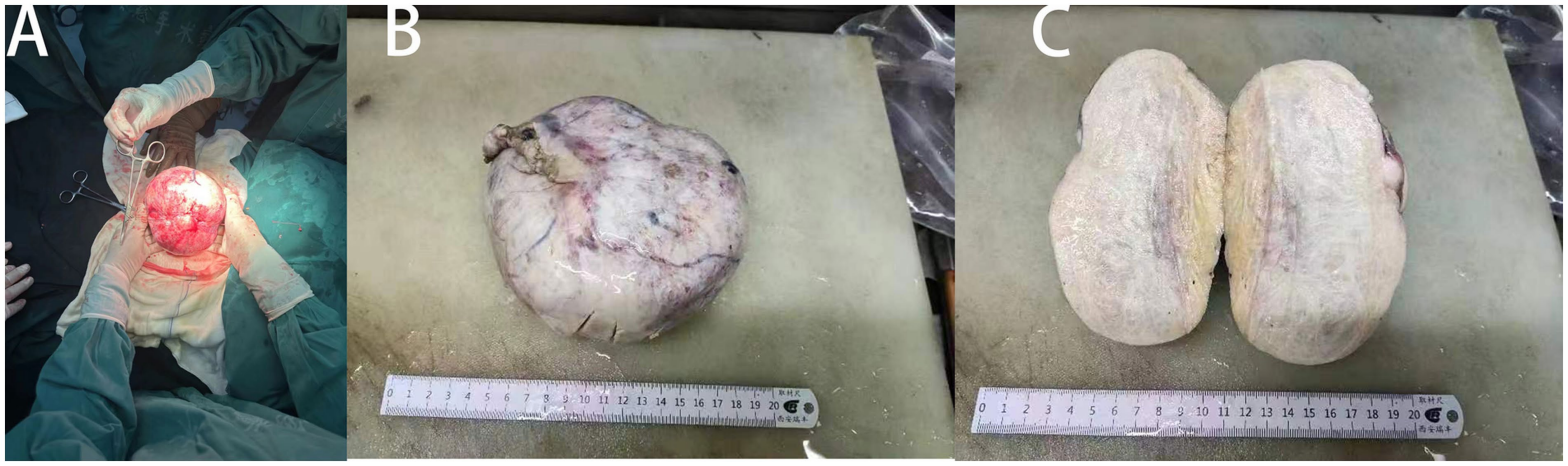
Intraoperative images of the patient’s lesions. **(A)** Image of ovarian tumor before removal. **(B)** Gross image after the removal of ovarian tumor. **(C)** Ovarian tumor profile.

This report describes a case in which intravenous general anesthesia, combined with ultrasound-guided TAP block-assisted analgesia, was used successfully for open exploratory abdominal surgery in a patient with ovarian tumors, hypertension, and significant ascites (see Figures 1, 2).

## Case report

A 63-year-old woman (163 cm, 78 kg) presented with progressive abdominal distension. Her medical history included uterine fibroids and grade II hypertension, with a maximum recorded blood pressure of 160/105 mmHg, which was treated with valsartan. Clinical examination revealed abdominal distension with increased wall tension, turbid percussion sounds, positive shifting dullness, and mild lower extremity edema. During hospitalization, the patient remained hemodynamically stable, with blood pressure ranging from 127 to 157/80 to 115 mmHg. Routine laboratory tests, including complete blood count, coagulation profile, and electrolytes, were unremarkable. Biochemical analyses showed hypoalbuminemia (albumin: 32 g/L and total protein: 55 g/L), elevated CA-125 level of 3,133 U/mL, and a thyroid-stimulating hormone (TSH) level of 9.06 μIU/mL. The cardiac evaluation revealed sinus tachycardia with ST-T abnormalities on electrocardiography and an ejection fraction of 70% on echocardiography, along with ventricular septal thickening and degenerative valvular changes. Imaging studies identified significant ascites, a pelvic mass, and associated findings, including hepatic cysts, right renal stones, uterine effusion, and bilateral pleural effusion. Gynecologic ultrasound suggested a follicular membranous cell tumor, while thoracic and abdominal CT showed coarse bilateral lung textures and irregular soft tissue density in the pelvis. A malignant pelvic tumor was strongly suspected, with an anticipated prolonged surgical duration. The combination of extensive ascites and hypertension presented the risks of intraoperative ventilation and circulatory instability, necessitating careful anesthetic planning. Therefore, the decision was made to administer intravenous general anesthesia in combination with ultrasound-guided TAP block-assisted analgesia to optimize hemodynamic stability.

The patient and her family were counseled preoperatively about the anesthetic plan, including the TAP block technique, its potential risks, and associated costs. Informed consent was obtained.

On the day of surgery, the patient was positioned supine in the operating room, and oxygen supplementation was initiated through a face mask. Routine monitoring was established, including electrocardiography, non-invasive blood pressure, pulse oximetry, bispectral index (BIS), and capnography. Lactated Ringer’s solution was infused through intravenous access. Sedation was induced by administering midazolam (2 mg) and etomidate (5 mg). A bilateral TAP block was performed under ultrasound guidance, with 30 mL of 0.2% levobupivacaine injected on each side. The sensory block was confirmed 20 min later using needle testing, with the patient reporting no discomfort. General anesthesia was induced with etomidate (15 mg), vecuronium bromide (6 mg), and sufentanil (10 μg), followed by tracheal intubation. Maintenance anesthesia was provided with continuous infusions of propofol (20 mL/h) and remifentanil (3 mL/h). Vasoactive agents, including dopamine and atropine, were prepared as needed.

Surgery began at 09:05, with ascitic fluid drained incrementally in four sessions, totaling 9,000 mL over 45 min. Throughout the procedure, hemodynamic stability was maintained, with blood pressure ranging from 90/56 to 132/70 mmHg and heart rates between 76 and 99 bpm. Prophylactic dopamine (2 mg) was administered during the initial drainage to mitigate decompression-induced hypotension. At 10:00, brief episodes of atrioventricular block lasting 3–5 s were observed, with a minimum heart rate of 45 bpm. These episodes resolved after a single intravenous dose of atropine (0.3 mg), and no recurrence was noted. Blood gas analysis at 10:22 showed normal findings: pH 7.44, PCO₂ 36 mmHg, PO₂ 128 mmHg, potassium 3.5 mmol/L, sodium 137 mmol/L, calcium 1.13 mmol/L, glucose 5.8 mmol/L, and lactate 0.6 mmol/L. The surgery proceeded with a transabdominal hysterectomy and bilateral adnexectomy. Specimens included an 8 × 5.5 × 4-cm uterus, a 3.5 × 1 × 0.5-cm right ovary, and a 13 × 13 × 7-cm left ovarian mass. Intraoperative rapid cryopathological analysis confirmed a benign ovarian interstitial tumor. The operation concluded at 11:45, and the patient was extubated at 11:55 after regaining full consciousness. The total surgical duration was approximately 1 h and 40 min. Postoperative pain was assessed using a visual analog scale (VAS) over 2 days, with the patient reporting minimal pain and no adverse effects, such as nausea, vomiting, or respiratory depression. Overall, the patient expressed high satisfaction with the anesthesia protocol.

## Discussion

With the global aging population on the rise, the demand for surgical treatments in older patients is increasing. However, these patients often have multiple comorbidities, which elevate the risks associated with perioperative anesthesia. Effective perioperative pain management is crucial for mitigating surgery-induced stress, minimizing postoperative complications, and promoting faster recovery.

The TAP block has become an integral component of multimodal analgesia in various abdominal procedures, including cesarean delivery, appendectomy, total abdominal hysterectomy, and laparoscopic cholecystectomy. Numerous studies have highlighted its effectiveness in reducing intraoperative and postoperative opioid consumption, lowering pain scores, and decreasing opioid-related side effects (5). For instance, a randomized controlled trial comparing bupivacaine and saline TAP blocks in patients undergoing cesarean delivery showed a > 60% reduction in total morphine consumption in the bupivacaine group, along with improved postoperative pain relief and fewer side effects, such as nausea and vomiting (6). Similarly, a study on laparoscopic cholecystectomy showed that patients who received TAP blocks experienced significantly lower postoperative pain scores, reduced analgesic requirements, and a lower incidence of nausea and vomiting than controls (4). Moreover, TAP blockade plays a key role in maintaining intraoperative hemodynamic stability. In a comparative study of anesthesia techniques, patients who received TAP block with the general anesthesia group exhibited fewer blood pressure fluctuations and a reduced need for phenylephrine than those who received general anesthesia alone or combined with epidural anesthesia (7). Another trial involving open gastric cancer surgery found that ropivacaine TAP blockade significantly lowered intraoperative systolic and diastolic blood pressure, heart rate fluctuations, and remifentanil use compared to saline TAP blockade (8). These findings highlight the role of TAP blockade in stabilizing vital parameters during surgery.

Ascites, characterized by the pathological accumulation of fluid in the peritoneal cavity, is typically classified into portal hypertensive ascites, non-portal hypertensive ascites, and mixed ascites (9). Ascites, which is frequently associated with cirrhosis and malignancies (10), causes a variety of debilitating symptoms, including dyspnea, abdominal pain, nausea, anorexia, and fatigue, all of which significantly impair quality of life (11). In the case presented, an older patient with an ovarian tumor and massive ascites faced several physiological challenges. Ascites-induced compression of the abdominal vasculature worsens afterload, reduces venous return, and increases the risk of lower extremity thrombosis due to venous stasis. Diaphragmatic elevation further compromises ventilation, leading to hypoxia and hypercapnia. In addition, patients with ascites frequently exhibit malnutrition, anemia, hypoproteinemia, and electrolyte imbalances, necessitating a thorough preoperative evaluation and tailored anesthetic strategies.

Given the patient’s condition, the dosage of general anesthesia was carefully reduced to prevent hemodynamic fluctuations during ascites drainage and tumor manipulation. To optimize analgesia and enhance perioperative stability, a multimodal approach combining general anesthesia with regional nerve blockade was used. Among the commonly used techniques, including TAP, erector spinae, and quadratus lumborum blocks, TAP blockade was preferred due to the patient’s abdominal distension, massive ascites, and the ability to perform the block in the supine position. Studies have shown that these techniques provide comparable analgesic outcomes and opioid-sparing effects (12, 13). This multimodal regimen yielded excellent outcomes. First, the combination of TAP block with anesthesia induction, using 20 mg etomidate, 2 mg midazolam, and 10 μg sufentanil, effectively provided analgesia. Second, the patient’s hemodynamics remained stable throughout ascites drainage, with no significant fluctuations in blood pressure or heart rate. The vasoactive agents, including dobutamine and atropine, were not required, except for a single prophylactic dose of 2 mg dobutamine to counteract decompression-induced hypotension. Finally, the patient experienced adequate postoperative pain control without adverse effects and expressed high satisfaction with the anesthetic technique.

In conclusion, ultrasound-guided TAP blockade provides substantial benefits as part of a multimodal analgesic strategy for patients with massive ascites. This approach reduces the need for anesthetic drugs, stabilizes intraoperative hemodynamics, and minimizes postoperative complications, ultimately improving patient outcomes and satisfaction. Advances in ultrasound technology have further improved the safety and applicability of TAP block, reinforcing its role in the perioperative management of complex cases such as this one.

## Data Availability

The original contributions presented in the study are included in the article/Supplementary material, further inquiries can be directed to the corresponding authors.

## References

[1] Rafi AN. Abdominal field block: a new approach via the lumbar triangle. Anaesthesia. (2001) 56:1024–6. doi: 10.1111/j.1365-2044.2001.2279-40.x, PMID: 11576144

[2] Brogi E Kazan R Cyr S Giunta F Hemmerling TM. Transversus abdominal plane block for postoperative analgesia: a systematic review and Meta-analysis of randomized-controlled trials. Can J Anaesth. (2016) 63:1184–96. doi: 10.1007/s12630-016-0679-x, PMID: 27307177

[3] Filardi K Filardi R Wegner B Arias J da Silva G Felippe V. Ultrasound-guided Transversus abdominis plane block as an effective path to reduce opioid consumption after laparoscopic bariatric surgery: a systematic review and meta-analysis of randomized controlled trials. Obes Surg. (2024) 34:4244–54. doi: 10.1007/s11695-024-07532-7, PMID: 39384705

[4] Emile SH Elfeki H Elbahrawy K Sakr A Shalaby M. Ultrasound-guided versus laparoscopic-guided subcostal Transversus abdominis plane (tap) block versus no tap block in laparoscopic cholecystectomy; a randomized double-blind controlled trial. Int J Surg. (2022) 101:106639. doi: 10.1016/j.ijsu.2022.106639, PMID: 35487422

[5] Young MJ Gorlin AW Modest VE Quraishi SA. Clinical implications of the Transversus abdominis plane block in adults. Anesthesiol Res Pract. (2012) 2012:731645:1–11. doi: 10.1155/2012/731645, PMID: 22312327 PMC3270549

[6] Baaj JM Alsatli RA Majaj HA Babay ZA Thallaj AK. Efficacy of ultrasound-guided Transversus abdominis plane (tap) block for postcesarean section delivery analgesia--a double-blind, placebo-controlled, randomized study. Middle East J Anaesthesiol. (2010) 20:821–6. PMID: 21526667

[7] Furuya A Ikemoto K Asano N Tamaki F Suzuki S Nonaka A. Assessment of intraoperative hemodynamics, infusion volume, urinary output and dose of circulatory drugs in general anesthesia with Transversus abdominis plane block for cholecystectomy. Masui. (2013) 62:1106–11. PMID: 24063137

[8] Li K Li L Gao M Zhu Z Chen P Yang L. Application of ultrasound-guided subcostal Transversus abdominis plane block in gastric cancer patients undergoing open gastrectomy. Int J Clin Exp Med. (2015) 8:13976–82. PMID: 26550355 PMC4613040

[9] Du L Zhu S Lu Z Xu T Bai T Xu D. Ascitic cholesterol is superior to serum-ascites albumin gradient in the detection of non-portal hypertensive ascites and the diagnosis of mixed ascites. Aliment Pharmacol Ther. (2019) 49:91–8. doi: 10.1111/apt.15042, PMID: 30443960

[10] Kipps E Tan DS Kaye SB. Meeting the challenge of ascites in ovarian Cancer: new avenues for therapy and research. Nat Rev Cancer. (2013) 13:273–82. doi: 10.1038/nrc3432, PMID: 23426401 PMC4673904

[11] Ford CE Werner B Hacker NF Warton K. The untapped potential of ascites in ovarian Cancer research and treatment. Br J Cancer. (2020) 123:9–16. doi: 10.1038/s41416-020-0875-x, PMID: 32382112 PMC7341795

[12] Warner M Yeap YL Rigueiro G Zhang P Kasper K. Erector Spinae plane block versus Transversus abdominis plane block in laparoscopic hysterectomy. Pain Manage. (2022) 12:907–16. doi: 10.2217/pmt-2022-0037, PMID: 36214314

[13] Sertcakacilar G Yildiz GO. Analgesic efficacy of ultrasound-guided Transversus abdominis plane block and lateral approach Quadratus Lumborum block after laparoscopic appendectomy: a randomized controlled trial. Ann Med Surg. (2022) 79:104002. doi: 10.1016/j.amsu.2022.104002, PMID: 35860161 PMC9289327

